# In Vitro Response of Human Peripheral Blood Mononuclear Cells (PBMC) to Collagen Films Treated with Cold Plasma

**DOI:** 10.3390/polym9070254

**Published:** 2017-06-29

**Authors:** Rui Chen, Jude Curran, Fanrong Pu, Zhuola Zhuola, Yves Bayon, John A. Hunt

**Affiliations:** 1 Department of Mechanical, Materials and Aerospace, School of Engineering, University of Liverpool, Harrison Hughes Building, Liverpool L69 3GH, UK; jmerice@liverpool.ac.uk (J.C.); zhuola@liverpool.ac.uk (Z.Z.); 2 Institute of Ageing and Chronic Disease, William Henry Duncan Building, University of Liverpool, Liverpool L7 8TX, UK; frpu1984@gmail.com (F.P.); john.hunt@ntu.ac.uk (J.A.H.); 3 Medtronic—Sofradim Production, 116 Avenue du Formans—BP132, F-01600 Trevoux, France; yves.bayon@medtronic.com; 4 CELS Building, School of Science and Technology, Nottingham Trent University, Nottingham NG11 8NS, UK

**Keywords:** plasma treatment, biocompatibility, inflammation, wound healing, nanotopography

## Abstract

The implantation of biomedical devices, including collagen-based implants, evokes an inflammatory response. Despite inflammation playing an important role in the early stages of wound healing, excessive and non-resolving inflammation may lead to the poor performance of biomaterial implants in some patients. Therefore, steps should be taken to control the level and duration of an inflammatory response. In this study, oxygen and nitrogen gas plasmas were employed to modify the surface of collagen film, with a view to modifying the surface properties of a substrate in order to induce changes to the inflammatory response, whilst maintaining the mechanical integrity of the underlying collagen film. The effects of cold plasma treatment and resultant changes to surface properties on the non-specific inflammatory response of the immune system was investigated in vitro in direct contact cell culture by the measurement of protein expression and cytokine production after one and four days of human peripheral blood mononuclear cell (PBMC) culture. The results indicated that compared to oxygen plasma, nitrogen plasma treatment produced an anti-inflammatory effect on the collagen film by reducing the initial activation of monocytes and macrophages, which led to a lower production of pro-inflammatory cytokines IL-1β and TNFα, and higher production of anti-inflammatory cytokine IL-10. This was attributed to the combination of the amino chemical group and the significant reduction in roughness associated with the introduction of the nitrogen plasma treatment, which had an effect on the levels of activation of the adherent cell population.

## 1. Introduction

Collagen, the main component of extracellular matrix, has not only been used as skin filler in cosmetics, wound dressing, sealants and adhesion barriers [1,2,3,4], it is also widely used to improve the biocompatibility of biomedical devices. Biomedical devices are made of synthetic polymers, metals and ceramics by physical adsorption or chemical grafting of collagen onto a surface [5,6,7,8,9]. The physical intervention of implantation into the human body always damages microvasculature and tissue, which evokes at least a non-specific inflammatory response [10,11]. Understanding and controlling the inflammatory response is a key requisite that will inform future biomaterial design. Inflammation not only helps clear out damaged and dead cells along with bacteria and other pathogens or foreign debris through the process of phagocytosis, it also recruits host cells for remodeling and regenerating the damaged tissue, and therefore can be exploited to aid tissue regeneration.

Despite inflammation playing an important role in the early stages of wound healing, excessive and non-resolving inflammation is one of the major factors that can ultimately result in the failure and rejection of biomaterial implants. A key performance requirement for these devices is the rapid stabilization of the device in the host to avoid persistent inflammatory stimuli, which leads to insufficient healing of local tissue at the surface of the medical device. Hence, it has long been an objective as a secondary function of biomaterials used for implanted medical devices to modulate or minimize excessive inflammation through minimizing fibrous tissue formation whilst encouraging the formation of the required de novo tissue [11,12]. Tailoring the surface properties of a biomaterial is a fundamental tool that can be used to control initial cellular responses, and thus play a fundamental role in controlling the inflammatory response.

The non-specific inflammatory response to implanted devices varies depending on their surface physicochemical properties, including surface chemistry, morphology and mechanics [12,13,14,15,16]. These properties affect the type, the quantity and the conformation of the adsorbed proteins, which determines the following interaction with adhesion receptors present on inflammatory cell populations, instigates a cascade of responses that are integral in determining the cellular driven inflammatory response, and constitutes the major cellular recognition system for implant materials [17,18].

Peripheral blood mononuclear cells (PBMCs) have been widely used as an in vitro model [10] to investigate biocompatibility and inflammation. The activation of the chronic inflammatory response is characterized by the presence of granulocytes, monocytes and possibly lymphocytes at the site of implantation [10,17]. Flow cytometry (FACS) analysis has demonstrated that PBMC layer isolation from whole blood via venipuncture is rich in both lymphocytes (T and B) and monocytes, which express CD3 (T lymphocyte marker), CD19 (B lymphocyte marker), CD14 (monocyte marker), and CD45 (generic leukocyte antigen used to label the whole population). Monocytes and macrophages are major cellular components that determine the severity and duration of the chronic inflammation [10]. Lymphocytes, especially T lymphocytes, modulate and/or enhance a monocyte-driven inflammatory response by increasing monocyte adherence to a material and their subsequent fusion to form foreign body giant cells (FBGC) [10,17,18]. PBMCs produce a wide array of cytokines in non-specific inflammatory response. A biomaterial may induce activation of monocytes/macrophages and lymphocytes to secrete pro-inflammatory cytokines, such as Interleukin-1β (IL-1β) and Tumor Necrosis Factor-α (TNF-α). Besides pro-inflammatory cytokines, PBMCs also secrete anti-inflammatory cytokines such as Interleukin-10 (IL-10), which when produced by monocytes/macrophages and some lymphocytes can inhibit the synthesis of pro-inflammatory cytokines produced by PBMC. Anderson and Jones [17,18] demonstrated that biomaterial surface chemistry could modulate the production of inflammatory molecules from monocytes/macrophages and lymphocytes in direct contact with biomaterials. Surface chemistry, surface morphology and roughness may modulate protein adsorption, leucocyte adhesion and inflammatory cytokine secretion [15,16,19,20,21]. Therefore, when blood cells are used for the investigation of the inflammatory response to materials, it is important to quantify cell adherence of both monocytes and lymphocytes and also to analyze the production and the release of cytokines.

In the last several decades, many surface modification technologies have been introduced into biomaterial design and manufacture to improve the biocompatibility of the materials [19]. It has been proven that cold plasma treatment is an effective method of modifying material surfaces without affecting bulk properties [22,23]. This process involves exciting gases such as oxygen (O_2_), nitrogen (N_2_), ammonia (NH_3_), tetrafluoromethane (CF_4_), chlorine (Cl_2_) or argon into an energetic state using electrons, radio frequency or microwaves. This then forms a layer of plasma, a partially ionized gas containing highly excited free radicals, atoms, electrons and ions, which produce unique physical and chemical surface properties [22,23,24]. Plasma treatment has been widely used to improve cell adhesion on biomaterial surfaces. Esposito et al. [25] proved that both oxygen and nitrogen plasma treatment increased hydrophilicity and roughness of PLGA samples, which were beneficial to cell growth by improving cell-polymer interaction. Lopez-Garcia et al. [26] demonstrated argon plasma treatment improved HaCaT keratinocyte proliferation on collagen films. As previously reported by our group, cold oxygen, nitrogen, argon and ammonia plasma treatment changed the chemical composition and morphology of biomaterial surfaces, which lead to the modulation of the protein adsorption and cell attachment [27,28,29].

Cold plasma has also been employed in wound healing [30] and sterilization [31]. The cold plasma treatment of cells has been reported to lead to their regeneration and rejuvenation, suggesting a plasma therapy program could be developed to help wound healing [30]. However, the ability of plasma treatment to promote cell attachment and proliferation may have adverse or beneficial inflammatory responses when those implants come into contact with blood and are involved in determining the cell-driven inflammatory response.

Until now, most plasma treatment research has focused on improving the cell attachment, proliferation and differentiation, but the effects of plasma treatment on inflammatory response are still not clear. In our previous study, we demonstrated that the surface properties of a biomaterial determined the types of cytokines secreted by leucocytes, and as a result will affect the inflammatory response to a particular biomaterial [10,22,32,33]. Therefore, if the surface characteristics of a biomaterial can be modified by cold plasma treatment to control the inflammatory response, this will result in improved outcomes once the material is implanted as part of a medical device. In this study, we investigated the effects of oxygen and nitrogen plasma treatment on the surface properties (hydrophobicity, morphology and roughness) of collagen biomaterials, and their effects on the relative amounts of cell surface protein expression and cytokine production after one and four days of direct contact cell culture with PBMC to understand interactions between the host immune system and plasma treated collagen biomaterials. The results also informed us as to the suitability of the plasma-treated biomaterials for further investigation and eventually implantation as part of a biomedical device.

## 2. Materials and Methods

### 2.1. Collagen Film Preparation and Plasma Treatment

Collagen solution was prepared from a solution of oxidized porcine atelocollagen type I (Sofradim Production, Trevous, France) and glycerol [7] and then spin-coating on a clean glass coverslip. Briefly, 1.8 mL 10% glycerol solution was added into 20 mL of oxidized bovine atelocollagen type I solution. The mixed solution was adjusted to pH = 7.0 by dropped 5% NaOH. 100 µL of collagen solution was pipetted onto 13 mm diameter clean coverslips mounted on a WS-400B-6NPP-Lite Single Wafer Spin Processor (Laurel Technologies Corporation, North Wales, PA, USA). After spinning at 1000 rpm, 2000 rpm, 3000 rpm, 4000 rpm and finally 2000 rpm for 15 s respectively, the collagen was observed to be evenly spread in a thin film and the membranes were left to dry for 24 h at room temperature in the fume hood.

Collagen-coated coverslips were then placed in 24-well plates within a plasma asher (Emitech K1050X, Quorum Technologies, Laughton, UK) and treated with either oxygen or nitrogen plasma at a pressure of 0.6 mbar with a flow rate of 15 mL/min at 80 W for 2 min. After treatment, specimens were returned to atmospheric pressure by venting with treatment gas. Treated materials were stored in sealed airtight container for two weeks in order to stabilize the post-treatment reactions.

### 2.2. In Vitro Experiment with Peripheral Blood Mononuclear Cell (PBMC)

#### 2.2.1. PBMC Isolation

Primary human mononuclear cells were isolated from heparinized human peripheral whole blood using previously published methods [32]. Briefly, 30 mL of whole blood was obtained via venipuncture from four healthy volunteers (each donor formed the basis of a single repeat) and anti-coagulated with 150 µL heparin (1000 iu/mL, CP Pharmaceutical Ltd., Wrexham, UK) at the Royal Liverpool University Hospital Phlebotomy Department as approved by the Liverpool Research Ethics Committee (LREC, reference number-02/06/084/A). Anticoagulated blood was layered onto 15 mL Histopaque-1077 (Sigma-Aldrich, Gillingham, UK) and centrifuged at 2000 rpm for 20 min at 4 °C (Sanyo MSE Mistral 3000i, Newport Pagnell, UK). The PBMC layer was removed using a Pasteur pipette and added to 30 mL PBS, which was centrifuged to obtain a cell pellet (1500 rpm, 10 min, 4 °C) (HERMLE Z513K, HermLe Labourtechnik, Wehingen, Germany). Cell counting was performed using a haemocytometer. 20 mL of PBS was added and cell washing by centrifugation was repeated. The supernatant was removed and cells were re-suspended in Roswell Park Memorial Institute-1640 (RPMI) medium (Gibco, Life Technologies, Inc., Paisley, UK) supplemented with 10% foetal calf serum (FCS) (Life Technologies, Inc., Paisley, UK), L-glutamine (15 mM) (Sigma-Aldrich, Gillingham, UK) and penicillin-streptomycin (5000 units/mL penicillin; 5 mg/mL streptomycin) (Sigma-Aldrich, Gillingham, UK).

#### 2.2.2 PBMC Culture on Collagen Biomaterials

Prior to cell seeding, all the materials were sterilized with 70% ethanol and washed with phosphate-buffered saline (PBS) (0.01 M Sigma-Aldrich). PBMCs (2.0 × 10^6^ cells/mL) were seeded onto collagen films with or without plasma treatments and incubated at 5% CO_2_ and 37 °C. The same numbers of wells were prepared for a positive control group, containing no collagen but stimulated by adding 25 ng/mL phorbol 12-myristate 13-acetate (PMA) (Sigma-Aldrich, Gillingham, UK) to the wells before incubation.

For every donor, each experiment consisted of 12 samples for each biomaterial type (collagen, positive control, oxygen and nitrogen plasma treated), six of which were investigated after one day and six after four days of culture.

### 2.3. Characterization of Materials Surface

#### 2.3.1. Advancing Contact Angle

Dynamic contact angles of the samples in deionized purified water were determined using the Wilhelmy plate method. Briefly, the contact angles for all the samples were determined using a Dynamic Contact Angle Tensiometer (CDCA 100, Camtel Ltd., Royston, and Herts, UK) at 22 ± 0.5 °C. Each sample was immersed into deionized water at a rate of 0.060 mm/s. The wetting force at the solid/liquid/vapor interface was automatically recorded as a function of both time and immersion depth, and was converted into advancing contact angles. The mean ± standard deviation of three samples for each material was reported, statistical analysis was confirmed using a student *t*-test to distinguish statistical significance, *p* = 0.05.

#### 2.3.2. Scanning Electron Microscopy (SEM)

The surface microstructures and profiles were observed using Field Emission Scanning Electron Microscopy (FE-SEM) (LEO 1550, Cambridge, UK). Briefly, the discs for SEM were coated with chromium (2 min and about 50 nm thick) under 125 mA. The coated sample was placed in the vacuum chamber of the SEM and scanned at a voltage of 5 kV. Samples were interrogated across the entirety of the surface and representative images are shown.

#### 2.3.3. Atomic Force Microscopy (AFM)

Atomic Force Microscopy (AFM) imaging was carried out using a commercial AFM (Dimension Icon, Bruker Co., Santa Barbara, CA, USA). The ScanAsyst mode was applied using a silicon tip (TAP150A, Bruker, nominal frequency of 150 kHz, nominal spring constant of 5 N/m) with a scan resolution of 512 samples per line at a scan rate of 1.0 Hz for an area 2.5 µm × 2.5 µm. Integral and proportional gains were optimized empirically during scanning. All post-image analysis was carried out using the built-in AFM software and Nanoscope Analysis (NanoScope VIII MultiMode AFM, Bruker Nano Inc., Nano Surfaces Division, Santa Barbara, CA, USA). Three randomly selected areas were scanned per sample and the results of roughness (root mean squared roughness, Rq) were presented as the mean ± standard deviation of three different samples for each group. The statistics used for contact angle analysis was a student *t*-test to distinguish statistical significance, *p* = 0.05.

### 2.4. Analysis of PBMCs Cultured on Collagen Biomaterials

#### 2.4.1. Cell Morphology and Cell Proliferation

At Day 1 and Day 4, cell attachment in all groups was visualized using light microscopy and images were taken to analyze differences in cellular morphology between the experimental groups (Axiovert 200, AxioVision Release 4.8.1 software, Carl Zeiss, Göttingen, Germany).

The samples were then washed with PBS, put into new wells with PBS and stored at −80 °C prior to cell viability assays. In order to obtain the cell numbers of leukocytes, cell counting was performed using commercially available CyQUANT cell proliferation assay (Molecular Probes, Eugene, OR, USA). The basis for the CyQUANT assay was the use of a proprietary green fluorescent dye (CyQUANT GR dye) that exhibits strong fluorescence enhancement when bound to cellular nucleic acids. Cells were lysed with a buffer containing CyQUANT GR dye. Fluorescence was measured using an FLX800 fluorimeter with excitation at 485 nm and emission at 530 nm. Sample fluorescence values were converted into cell numbers from a standard curve by measuring the fluorescence of a set of samples of known cell number ranging from 100,000 to 50 cells. A total of three separate repeats were carried out for each experimental group at Day 1 and Day 4 per donor (donor number = 4). Results are shown as mean ± standard deviation and ANOVA Tukey and Waller Duncan models were used to distinguish statistical significance, *p* = 0.05.

#### 2.4.2. Immunofluorescence Flow Cytometry

Cells adhered to the collagen films were detached by trypsinization and shear force washing using PBS. Three wells in each experimental group were combined and centrifuged (1500 rpm, 5 min) to obtain a cell pellet. Supernatant was removed and the cells were re-suspended in 250 µL of FACS Flow fluid (BD Biosciences, Oxford, UK) containing 2% FCS. Cells were incubated for 30 min at 4 °C with fluorescein isothiocyanate (FITC) conjugated mouse anti-human monoclonal antibodies against CD3 (T lymphocyte marker, BD Biosciences, Oxford, UK), phycoerythrin (PE) conjugated mouse anti-human monoclonal antibodies against CD19 (B lymphocyte marker, BD Biosciences, Oxford, UK), phycoerythrin-Cy5 (PE-Cy5) conjugated mouse anti-human monoclonal antibodies against CD45 (generic leukocyte antigen used to label the whole population, BD Biosciences, Oxford, UK), and fluorescein isothiocyanate (FITC) conjugated mouse anti-human monoclonal antibodies against CD14 (monocyte marker, AbD Serotec, Oxford, UK). Appropriate IgG1-κ isotype control antibody conjugates against FITC, PE and PE-Cy5 (BD Biosciences, Oxford, UK) established the level of background fluorescence. Cells were fixed in 150 µL of CellFix (BD Biosciences, Oxford, UK) and stored at 4 °C until analyzed. 230 µL of FACSFlow/2% FCS was added to cells and a total of 20,000 events were collected using a FACSort flow cytometer and CellQuest software (BD, San Jose, CA, USA). Results were expressed as the relative proportions of positive expression of each antibody (over and above the isotype control levels for each fluorophore) in the total sample of gated events. This process was performed after one and four days of culture. Results are shown as mean ± standard deviation. ANOVA Tukey and Waller Duncan models were incorporated to establish the statistically significance of the results, *p* = 0.05, for a total of three replicates for each CD antigen of each experimental group per donor (donor number = 4).

#### 2.4.3. Pro/anti-Inflammatory Cytokine Release

The supernatants from in vitro cell culture experiments were collected and stored at −20 °C for ELISA analysis. Quantification of human IL-1β, TNF-α and IL-10 were achieved using commercially available kits (Invitrogen™). Prior to assay, the designated cell culture supernatants were thawed at room temperature and placed into designated wells within a 96-well plate. All kit reagents were brought to room temperature and gently mixed without foaming. A total of four separate replicates were carried out for each of the cytokines of each sample group per donor at Day 1 and Day 4 (donor number = 4). Results are shown as mean ± standard deviation and ANOVA Tukey and Waller Duncan models were used to distinguish statistical significance, *p* = 0.05.

## 3. Results

### 3.1. Surface Characterization

**Figure 1:**
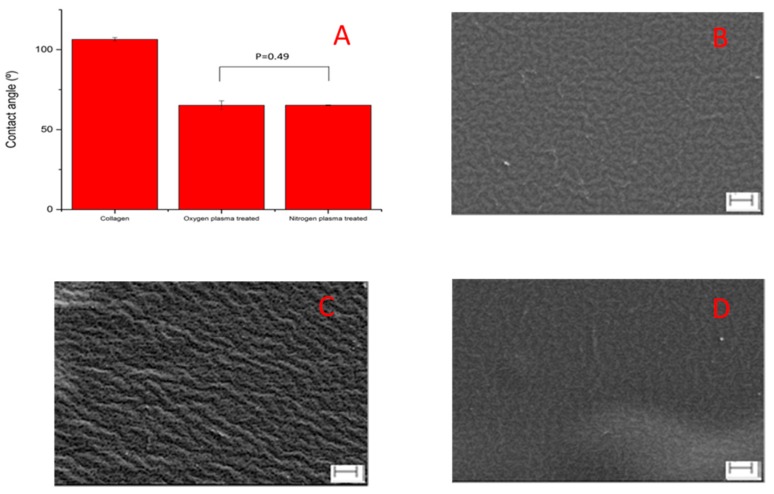
The advancing contact angle and Scanning Electron Microscopy (SEM) images of collagen films with/without oxygen and nitrogen plasma treatment. (**A**) advancing contact angle results (*n* = 3); (**B**) SEM image of untreated collagen film; (**C**) SEM image of oxygen plasma-treated collagen film; (**D**) SEM image of nitrogen plasma-treated collagen film. Scale bar = 2 µm. The SEM images showed many small pits were produced and the height of ridges increased after oxygen plasma treatment; the height of ridges decreased after nitrogen plasma treatment.

The advancing contact angle of untreated collagen film was 106.4 ± 1.1° (*n* = 3). After oxygen and nitrogen plasma treatment at 80 w for 2 min, the contact angle decreased to 65.2 ± 2.8° (*n* = 3) and 65.2 ± 0.3 (*n* = 3), respectively. There was no significant difference between the contact angles of these two treatments (Figure 1A). SEM (Figure 1B–D) showed that there were no distinct changes in surface morphologies after nitrogen plasma treatment. Many small pits were produced on the collagen film surface after oxygen plasma treatment because of the strong etching effect of oxygen plasma.

**Figure 2:**
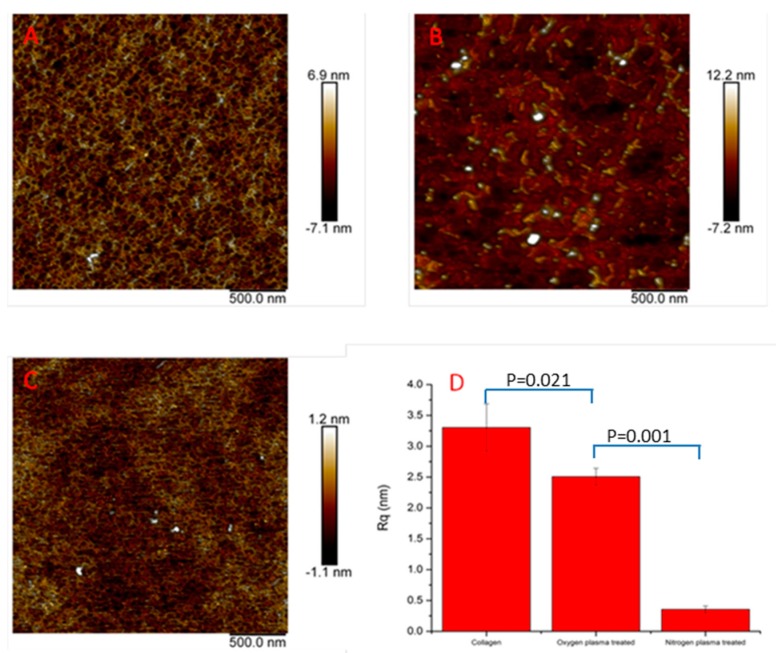
The results of Atomic Force Microscopy (AFM) morphology and roughness. (**A**) untreated collagen film; (**B**) oxygen plasma-treated collagen film; (**C**) nitrogen plasma-treated collagen film; (**D**) the root mean squared roughness results across 1.4 µm × 1.4 µm (*n* = 3, repeats = 3).

The results of AFM analysis (Figure 2) showed that the network structure of the collagen films was decomposed and many small ridges appeared on the surfaces after oxygen plasma treatment (Figure 2B). However, the network structure was maintained with nitrogen plasma treatment (Figure 2C). The roughness result (Figure 2D) showed that the roughness of collagen films decreased significantly from 3.3 ± 0.4 nm (*n* = 3) for untreated collagen film to 2.5 ± 1.4 nm (*n* = 3) for oxygen plasma-treated collagen film, and 0.35 ± 0.06 nm (*n* = 3) for nitrogen plasma-treated collagen film, which suggests that nitrogen plasma treatment made the surface significantly smoother and uniform. The roughness of the nitrogen plasma-treated samples was also significantly reduced when compared to the oxygen plasma treated samples.

### 3.2. PBMCs Attachment on the Collagen Surface

**Figure 3:**
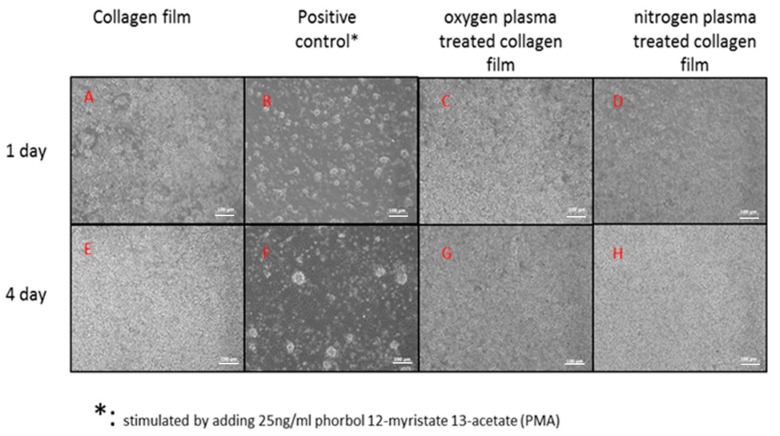
Micrographs of peripheral blood mononuclear cell (PBMC) cultured on biomaterial surfaces after one day (upper-row: (**A**–**D**)) and four days (down-row: (**E**–**H**)). Scale bar = 100 µm.

Qualitative evaluation of the adherent cell population (Figure 3) showed similar levels of homogenous cell coverage associated with the untreated collagen and nitrogen and oxygen plasma-treated samples. There were no distinguishable differences in cell morphology associated with the untreated (Figure 3A,E) and treated collagen samples (Figure 3C,D,G,H). Confluence across the surfaces was achieved by Day 4 in PBMC cultures. Comparatively fewer cells were present in the positive control membranes, which demonstrated activation of macrophages (cell clustering to form larger cells and presentations of roughened membranes) at one and four days in PBMC cultures (Figure 3).

**Figure 4:**
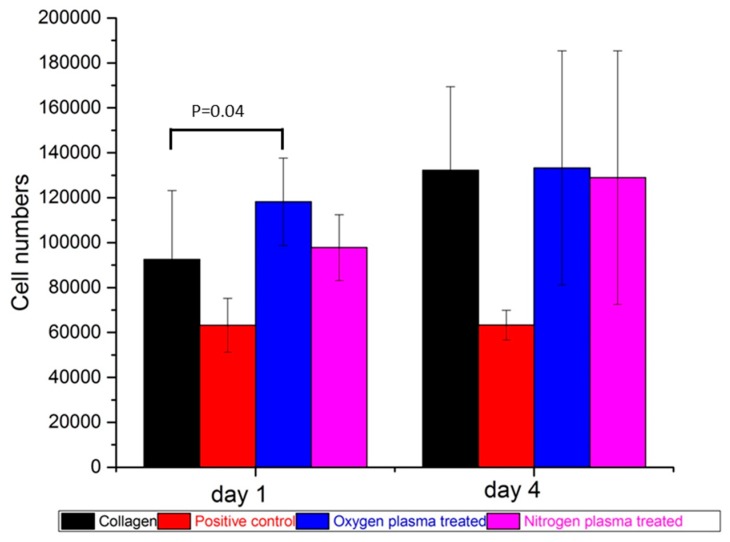
The number of cells attached on collagen, positive control, oxygen and nitrogen plasma-treated collagen films after PBMC cultured one day and four days. (Donors = 4, repeats = 3).

A quantitative valuation of viable cell numbers (Figure 4) showed that oxygen plasma treatments increased the PBMC attachment compared to untreated collagen (*p* = 0.04) at Day 1; and there was no statistical difference between oxygen and nitrogen plasma treatments’ collagen surface at Day 1. At Day 4, there were no distinct differences among the number of PBMC attached on untreated and plasma-treated surfaces. This was in line with the previously discussed qualitative evaluation shown in Figure 3.

### 3.3. Immunofluorescence Flow Cytometry Results

**Figure 5:**
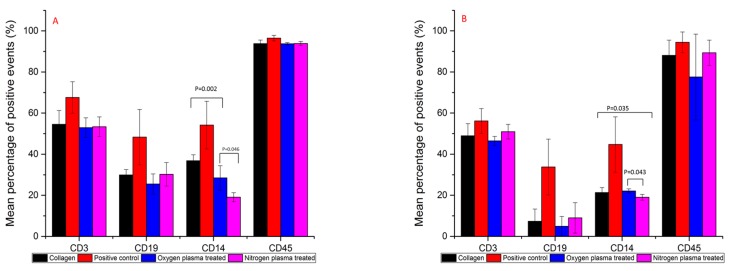
Mean relative percentage expression of CD antigens (CD3, CD19, CD14 and CD45) on collagen, positive control, oxygen plasma-treated and nitrogen plasma-treated collagen films after one day (**A**) and four days (**B**) of PBMC culture. (Donors = 4, repeats = 3).

Immunofluorescence flow cytometry results (Figure 5) showed that CD3 expression after one and four days of PBMC culture was not statistically significantly different between the collagen films, although the percentage of positive expression was highest in the positive control group in all repeats at both time points. CD19 expression was significantly higher in the positive control group than all other groups tested at Day 1 (*p* = 0.003 to *p* = 0.019) and Day 4 (*p* = 0.002 to 0.006). CD19 expression was significantly decreased from Day 1 culture to Day 4 (*p* = 0.00 to 0.001), which indicated that B cells detached from all the surfaces after four days of culture. Mean CD45 expression was significantly higher in the positive control group (96.5%) after one day of culture than the control, oxygen, and nitrogen-treated collagen films with mean expressions of 93.8%, 93.8%, 93.6% and 93.9%, respectively. Day four analysis showed there to be no statistical difference between the groups.

After one day of PBMC culture, the positive control sample produced a significantly higher percentage of CD14 expression than all other groups (*p* = 0.00 to 0.01). Expression was significantly lower in the nitrogen plasma-treated films compared to the control films (*p* = 0.002) and oxygen plasma-treated films (*p* = 0.046) at Day 1. After four days, CD14 expression remained significantly higher in the positive control group (*p* = 0.001 to 0.004). CD14 expression was still significantly lower in the nitrogen plasma-treated films compared to the control films (*p* = 0.035) and oxygen plasma-treated films (*p* = 0.043). The percentage of positive expression was similar between the nitrogen-treated samples after one and four days of culture (19.1% vs. 19.03%), whereas the control and oxygen treated films had lower CD14 expressions after four days of culture compared to Day 1, with mean values of 36.8% vs. 21.3% and 28.5% vs. 22.08%, respectively.

### 3.4. Pro/anti-Inflammatory Cytokine Release after **1** Day and **4** Days PBMC Culture

**Figure 6:**
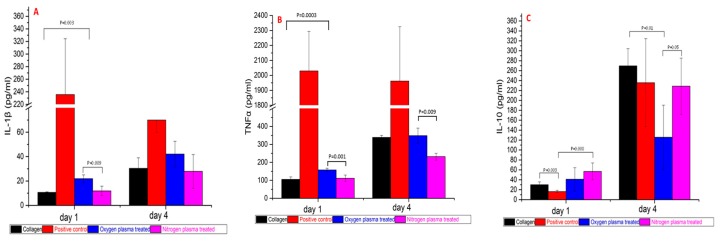
Cytokines production by PBMC cultured on collagen, positive, oxygen plasma-treated collagen and nitrogen plasma-treated collagen at Day 1 and Day 4. (**A**) IL-1 production; (**B**) TNFα production; (**C**) IL-10 production. (Donors = 4, repeats = 4).

The pro/anti-inflammatory cytokine release profiles after one day and four days PBMC culture (Figure 6) demonstrated that PBMC in the positive control group produced significant higher pro-inflammatory cytokines IL-1β and TNF-α at both Day 1 and Day 4 (*p* = 0.00 to 0.02). After one day of culture, PBMC produced more IL-1β on oxygen plasma-treated collagen film than untreated and nitrogen plasma-treated collagen film (*p* = 0.00 to 0.01); however, there was no significant difference at day four (*p* = 0.09 and 0.12). PBMC-produced TNF-α on collagen films showed the same trend as IL-1β. PBMC produced more TNF-α on oxygen plasma-treated collagen film than untreated and nitrogen plasma-treated collagen film (*p* = 0.00 to 0.01) at Day 1, and there was more TNF-α produced on oxygen plasma-treated collagen film than nitrogen plasma-treated collagen film at Day 4 (*p* = 0.009).

PBMC-produced anti-inflammatory cytokine (IL-10) increased on collagen film and nitrogen plasma-treated collagen film compared to the positive control group at Day 1 (*p* = 0.003, 0.008). All the groups produced more IL-10 at Day 4 compared to Day 1 (*p* = 0.00 to 0.01). However, PBMC on oxygen plasma-treated collagen film produced lower amounts of IL-10 than on collagen film and nitrogen plasma group at Day 4 (*p* = 0.01, 0.05).

## 4. Discussion

The effects of treating collagen films with or without cold oxygen and nitrogen plasma on the inflammatory response of human PBMC by direct contact were determined. Cold plasma treatment was an effective and economical surface modification technique, which has been widely used to modulate material surface properties without affecting bulk properties. During the cold plasma treatment, the electrons generated in the discharge gas impacted the surface with energies two to three times that which was necessary to break the molecular bonds on the surface of most substrates. This creates highly reactive free radicals, which in the presence of oxygen can react rapidly to form various more or less stable chemical functional groups (O-functionalities) on the substrate surface. These include carbonyl (–C=O), carboxyl (–COOH), hydrogen peroxide (–HOOH) and hydroxyl (–OH) groups [23,24,29]. Different to oxygen plasma treatment, nitrogen plasma treatments give rise to N-functionalities, such as amino (–NH_2_), imino (–C=N–), cyano (–C≡N) [24,29]. These polar functionalities made the surfaces more hydrophilic, and oxygen and nitrogen plasma provide similar results by decreasing of the contact angle (Figure 1). It was also demonstrated that the oxygen plasma treatment decomposed the 3D network structure at the nanoscale, whilst nitrogen plasma treatment maintained the 3D network structure of collagen films and made the surface significantly smoother compared to both the untreated and oxygen-treated collagen films (Rq decreased from 3.30 to 0.35 nm) (Figure 3D).

To evaluate the effects of surface properties on the inflammatory response of the immune system in vitro, the relative amounts of cell surface protein expression and cytokine production after one and four days of peripheral blood mononuclear cell (PBMC) culture were measured. PBMCs have been widely employed to test biocompatibility, as monocytes and macrophages play major roles in inflammation and are one of the key determinants of the success of an implanted biomaterial. Therefore, one of the main objectives of this study was to investigate whether the biomaterial surfaces produced different effects on activation of an adherent monocyte/macrophage population. The expression of CD14 was used to assess the response to the different biomaterials, as previous studies have shown it was upregulated after contact with non-compatible biomaterials [10,13,19]. As expected, the positive control group produced a significantly higher mean CD14 expression than all other biomaterials after one day of PBMC culture (*p* = 0.00 to 0.01). The nitrogen-treated film produced significantly lower CD14 expression than the control film (*p* = 0.009), suggesting that nitrogen plasma treatment reduced the number of activated monocytes and macrophages. The mean expression of CD14 after one day in the nitrogen treated sample was 19.0%, while expression in the collagen, positive control, and oxygen plasma treated samples were 36.8%, 54.2%, and 28.5%, respectively. After four days of PBMC culture, CD14 expression in the positive control group was still significantly higher than all other biomaterials (*p* = 0.001 to 0.004). The mean expression in the untreated collagen reduced to levels similar to the nitrogen plasma-treated sample at one day.

The other main objective of this research was to investigate the cytokine production and release in PBMC-biomaterials interaction, and use this to determine the potential inflammatory response. Cytokines are soluble factors that are mostly generated by immune cells that play crucial roles in the differentiation, maturation, and activation of various immune cells [10]. An analysis of pro/anti-inflammatory cytokines could provide a better understanding of the signaling process involved in the inflammatory response initiated by the different biomaterial surfaces. IL-1β enables transmigration of inflammatory cells to the site of implantation by increasing adhesion receptor expression on endothelial cells, and TNF-α activates phagocytic cells and stimulates the release of IL-1 and PGE2 [10]. For materials treated using oxygen plasma, the production of both IL-1β and TNFα increased at Day 1 compared to the untreated collagen and nitrogen plasma-treated materials; after nitrogen plasma treatment, production of IL-1β, TNFα decreased at Day 1 and Day 4 compared to untreated collagen film. IL-10 is an anti-inflammatory cytokine capable of inhibiting synthesis of pro-inflammatory cytokines such as IFN-γ, IL-2, IL-1β, TNFα and GM-CSF, which are made by cells such as macrophages and Th1 T cells [10,20]. It also displays a potent ability to suppress the antigen-presentation capacity of antigen-presenting cells. Due to a decrease in IL-10 levels in oxygen plasma-treated materials, TNFα levels were not regulated effectively as IL-10 regulates the TNF-α-converting enzyme [20]. As a result, TNFα levels rose and resulted in extensive inflammation in the oxygen plasma treated group.

The changes to the surface properties of collagen biomaterials induced by nitrogen plasma treatment resulted in a reduced initial reaction of monocytes and macrophages, which resulted in a lower production of the pro-inflammatory cytokines IL-1β and TNFα, and higher production of anti-inflammatory cytokine IL-10. Oxygen plasma was more effective in changing the surface properties of the collagen materials and resulted in an elevated initial reaction of monocytes and macrophages, a higher production of IL-1β, TNFα, and a lower production of IL-10.

The reason behind the different effects of oxygen and nitrogen plasma treatment on inflammatory response could be due to changes in the surface physio-chemical properties, including surface roughness and chemistry. Several studies have demonstrated that a variety of cell types are involved in the inflammatory response, including macrophages, leukocytes and granulocytes, which demonstrate adhesion on rough surfaces to a greater extent than smooth surfaces [15,16,21]. The smoother surface produced by nitrogen plasma treatment decreased cell adhesion and decreased physical stimulation of leucocytes. Besides the roughness, chemical groups present on the materials also affect cell activation. For example, hydroxyl groups introduced by oxygen plasma treatment strongly activate the complement system through the alternative pathway [34,35], which would increase macrophage activation; whereas amino groups introduced by nitrogen plasma treatment activate the complement cascade to a lesser extent [36].

## 5. Conclusions

Oxygen and nitrogen cold plasma treatment, performed at 80 W, 2 min at a pressure of 0.6 mbar with a flow rate of 15 mL/min on collagen films, modified material surfaces in not only their chemical composition and surface energy, but also their surface morphology. Oxygen plasma treatment decomposed the 3D collagen network structure and made the surface rougher at the nanoscale; nitrogen plasma treatment maintained the 3D network structure of collagen films and made the surface significantly smoother.

Nitrogen plasma treatment may impart an anti-inflammatory effect on collagen film by reducing initial activation of monocytes and macrophages, which can led to lower amounts of pro-inflammatory cytokines IL-1β and TNFα, and higher amounts of anti-inflammatory cytokine IL-10. This was attributed to the smoother surface and/or the amino chemical group introduced by nitrogen plasma treatment.
